# Supplementary material for: Mechanistic Investigation of the Androgen Receptor DNA-Binding Domain and Modulation via Direct Interactions with DNA Abasic Sites: Understanding the Mechanisms Involved in Castration-Resistant Prostate Cancer
Source: Int J Mol Sci. 2023 Jan 9;24(2):1270. doi: 10.3390/ijms24021270 (PMC9864221; doi:10.3390/ijms24021270)
Supplement: Supplementary file 1 [file ijms-24-01270-s001.zip › ijms-2100124-supplementary.pdf]

Mechanistic Investigation of the Androgen Receptor DNA-Binding Domain and Modulation via Direct Interactions with DNA Abasic Sites: Understanding the Mechanisms Involved in Castration-Resistant Prostate Cancer

Shangze Xu [1,2,†], Matthew D. Kondal [1,†], Ayaz Ahmad [1], Ruidi Zhu [1], Lanyu Fan [1,3], Piotr Zaborniak [1], Katrina S. Madden [1,4], João V. de Souza [1,*] and Agnieszka K. Bronowska [1,2,*]

1 Chemistry—School of Natural and Environmental Sciences, Newcastle University, Newcastle Upon Tyne NE1 7RU, UK
2 Newcastle University Centre for Cancer, Newcastle University, Newcastle Upon Tyne NE1 7RU, UK
3 School of Engineering, Newcastle University, Newcastle Upon Tyne NE1 7RU, UK
4 Translational and Clinical Research Institute, Newcastle University, Newcastle Upon Tyne NE2 4HH, UK
* Correspondence: joao-victor.de-souza-cunha@newcastle.ac.uk (J.V.d.S.); agnieszka.bronowska@ncl.ac.uk (A.K.B.).
† These authors contributed equally to this work.

Supplementary data

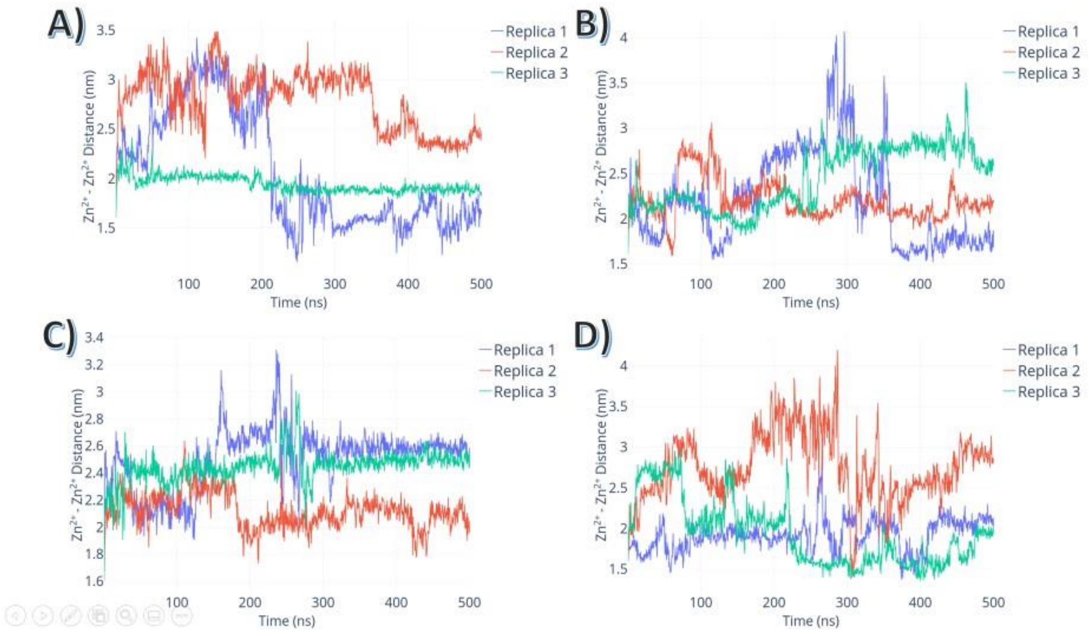

Figure S1. Inter-Zn^{2+} distance for mutant monomer runs: (**A**) A598S; (**B**) A597V; (**C**) L588P; (**D**) K591R.

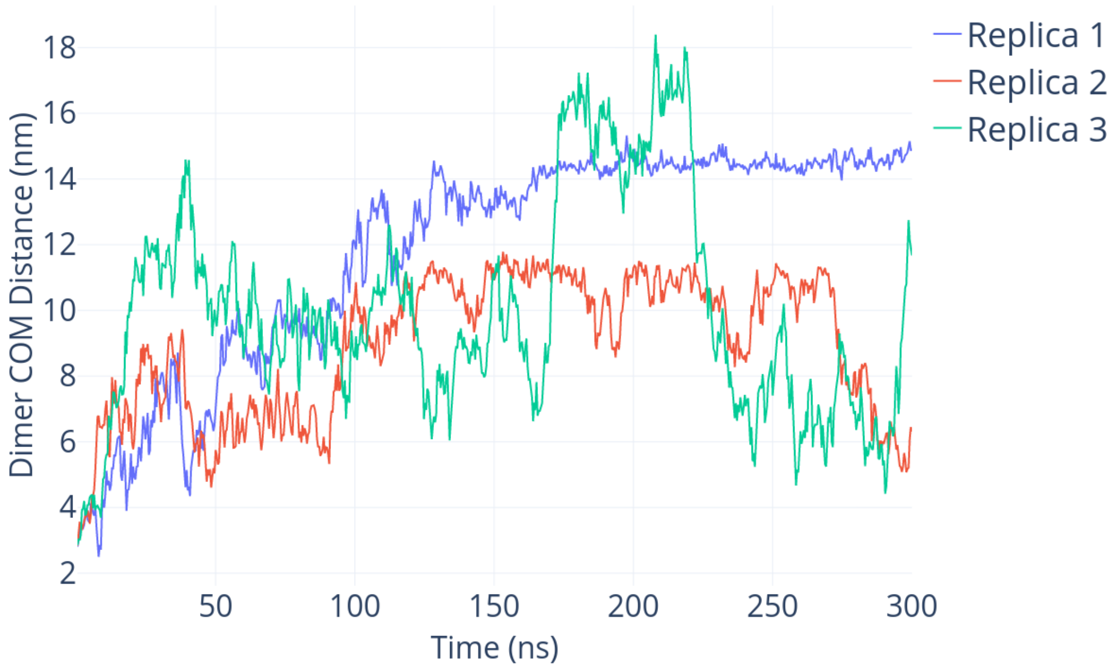

Figure S2. Distance between centre of mass of each monomer in a DNA-free simulations though the simulation time.
